# Intravital imaging reveals glomerular capillary distension and endothelial and immune cell activation early in Alport syndrome

**DOI:** 10.1172/jci.insight.152676

**Published:** 2022-01-11

**Authors:** Georgina Gyarmati, Urvi Nikhil Shroff, Audrey Izuhara, Xiaogang Hou, Stefano Da Sacco, Sargis Sedrakyan, Kevin V. Lemley, Kerstin Amann, Laura Perin, János Peti-Peterdi

**Affiliations:** 1 Department of Physiology and Neuroscience and Department of Medicine, Zilkha Neurogenetic Institute, and; 2 GOFARR Laboratory for Organ Regenerative Research and Cell Therapeutics in Urology, Children’s Hospital Los Angeles, Division of Urology, Saban Research Institute, University of Southern California, Los Angeles, California, USA.; 3 Division of Nephrology, Children’s Hospital Los Angeles, Los Angeles, California, USA and Department of Pediatrics, Keck School of Medicine, University of Southern California, Los Angeles, California, USA.; 4 Department of Nephropathology, Institute of Pathology, Friedrich-Alexander University, Erlangen, Germany.

**Keywords:** Nephrology, Collagens, Endothelial cells, Mouse models

## Abstract

Alport syndrome (AS) is a genetic disorder caused by mutations in type IV collagen that lead to defective glomerular basement membrane, glomerular filtration barrier (GFB) damage, and progressive chronic kidney disease. While the genetic basis of AS is well known, the molecular and cellular mechanistic details of disease pathogenesis have been elusive, hindering the development of mechanism-based therapies. Here, we performed intravital multiphoton imaging of the local kidney tissue microenvironment in a X-linked AS mouse model to directly visualize the major drivers of AS pathology. Severely distended glomerular capillaries and aneurysms were found accompanied by numerous microthrombi, increased glomerular endothelial surface layer (glycocalyx) and immune cell homing, GFB albumin leakage, glomerulosclerosis, and interstitial fibrosis by 5 months of age, with an intermediate phenotype at 2 months. Renal histology in mouse or patient tissues largely failed to detect capillary aberrations. Treatment of AS mice with hyaluronidase or the ACE inhibitor enalapril reduced the excess glomerular endothelial glycocalyx and blocked immune cell homing and GFB albumin leakage. This study identified central roles of glomerular mechanical forces and endothelial and immune cell activation early in AS, which could be therapeutically targeted to reduce mechanical strain and local tissue inflammation and improve kidney function.

## Graphical abstract

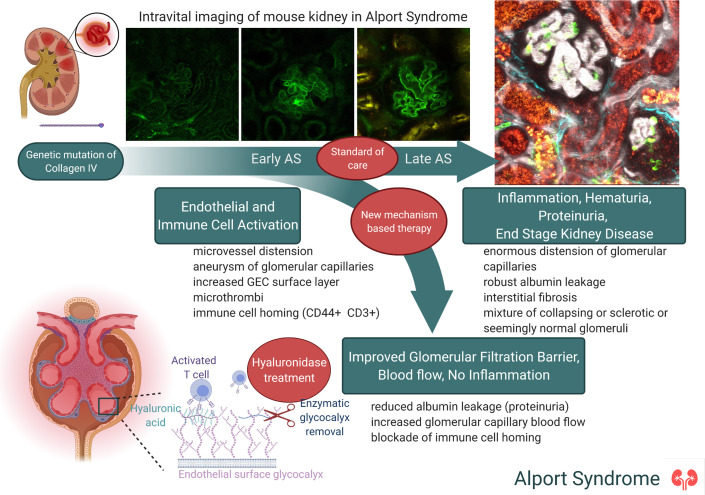

## Introduction

Alport syndrome (AS) is a hereditary form of chronic kidney disease (CKD) caused by mutations in one of the α chains of the collagen type IV trimer (*COL4A3/A4/A5*), which is a major constituent of the glomerular basement membrane (GBM) in the glomerular filtration barrier (GFB) (1). The defective GBM causes dysfunction and leakiness of the GFB, development of proteinuria, hematuria, and focal segmental glomerulosclerosis. Regardless of the type of genetic mutation (X-linked, autosomal recessive, or dominant AS) and despite diagnosis at an early age, many patients with AS develop progressive CKD and ultimately end-stage kidney disease (1). Although the various genetic mutations leading to AS are well known, mechanistic details of the disease pathogenesis have been elusive, hindering the development of mechanism-based therapies. While the GBM is abnormal already at the time of birth, glomerular function is initially normal, with a delayed onset of dysfunction and progressive disease (2). This observation has important implications about the mechanism of disease initiation. Because only podocytes produce COL4A3/A4/A5, podocyte dysfunction is considered the major culprit in the pathogenesis of CKD in AS (2). However, AS may not be only a podocyte-driven disease. Our recent work established the role of glomerular endothelial cells (GECs) in early AS (3), and GEC damage is known to precede proteinuria in multiple renal pathologies including AS (4, 5). A mechanistic understanding of AS development is important for the development of specific targeted therapeutic approaches to delay or prevent GFB injury and CKD. The nonspecific use of angiotensin-converting enzyme (ACE) inhibitors (ACEi) has been the gold-standard treatment to delay the progression of AS as well as other types of kidney diseases (6).

Structural and functional alterations in the local tissue microenvironment are major determinants of disease development and progression. Therefore, the ability to look inside intact living tissues may provide important mechanistic insights. Intravital imaging with multiphoton microscopy (MPM) is a state-of-the-art technique to visualize dynamic processes in vivo with subcellular resolution, and it has been used in many organs, including the kidney, to investigate disease pathogenesis (7–9). Recently, we used intravital MPM to study interactions between circulating activated T cells and GECs in the local kidney tissue microenvironment in lupus nephritis (10).

In this work, we applied similar in vivo imaging approaches to quantitatively visualize the altered structure and function of the glomerular capillaries — and specifically local GECs and immune cells — during the course of AS development. This focus is consistent with that used in our recently published work, which identified the roles of specific immune mechanisms (3) and GEC damage preceding proteinuria development in AS (4). In addition, we tested the therapeutic efficacy of enzymatic surface layer (glycocalyx) removal in AS, because the endothelial glycocalyx is the first point of contact between circulating immune cells and the local tissue environment during inflammation, and this treatment approach was found to be protective in experimental lupus nephritis (10).

## Results

### A view of AS pathology using intravital MPM imaging.

**Figure 1:**
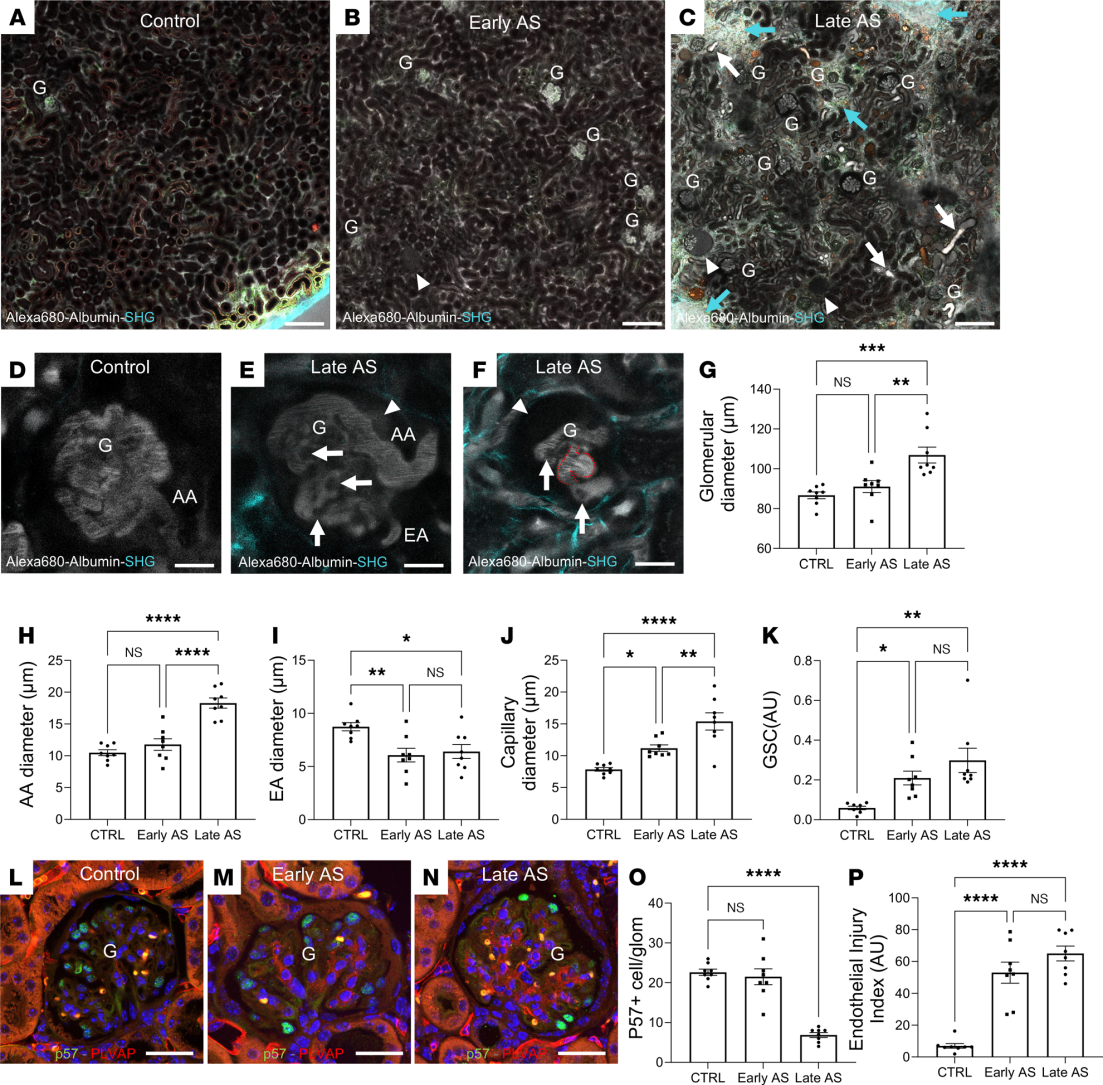
Overview MPM images and microvascular features of AS pathology in the intact living mouse kidney. (**A–C**) Overview of the renal cortex in 2-month-old control (**A**) and AS (Early AS; **B**) and 5-month-old AS mice (Late AS; **C**). Plasma was labeled with albumin–Alexa Fluor 680 (gray), and second harmonic generation (SHG; cyan) illuminated fibrotic tissue regions (cyan arrows). Optical sections were acquired approximately 150 μm under the kidney surface. Note the presence of normal glomeruli (no albumin filtration) among albumin-leaking and collapsing glomeruli (arrowheads; gray filtration space in **G** and tubular lumen) and tubular protein casts (white arrows) in AS mice (**B** and **C**) but not in controls (**A**). G, glomerulus. Scale bar: 100 μm. (**D–F**) Close-up images of glomeruli in late-stage AS mice (**E** and **F**) compared with controls (**D**) demonstrate severe distension of the afferent arteriole (AA) and glomerular capillaries, the presence of capillary aneurysms (red dotted line illustrates capillary wall), microthrombi and albumin-excluding dark cells (arrows) sticking to capillary walls and blocking red blood cell passage (dark bands in the light gray plasma), robust GFB albumin leakage (arrowheads), and interstitial fibrosis (SHG; cyan). EA, efferent arteriole. Scale bars: 20 μm. (**G–K**) Statistical summary of the changes in largest glomerular (**G**), afferent arteriole (**H**), efferent arteriole (**I**), and glomerular capillary diameters (**J**) and glomerular albumin leakage (albumin glomerular sieving coefficient [GSC]; **K**) in early- and late-stage AS mice compared with controls (CTRL). (**L–P**) Representative immunofluorescence images (**L–N**) and quantification (**O** and **P**) of the number of podocyte marker p57^+^ cells per glomerular section (green) and the PLVAP^+^ (glomerular endothelial injury marker; red) area of the glomerular tuft in control, early-stage, and late-stage AS mice. Nuclei are stained blue with DAPI. Scale bars: 20 μm. Data are shown as the mean ± SEM. *P < 0.05, **P < 0.01, ***P < 0.001, ****P < 0.0001, using 1-way ANOVA with Tukey’s multiple-comparison test. Data points represent the average of multiple measurements/mouse for *n* = 8 mice in each group.

Intravital MPM imaging of the intact kidney tissue microenvironment from early-stage (2 months old) and late-stage (5 months old) AS and healthy age-matched mice was performed to gain new in vivo visual clues regarding alterations in kidney structure and function and the potential major molecular and cellular mechanistic drivers at play during AS pathogenesis. An overview of the entire kidney cortex area using intravenously injected, fluorescently labeled albumin to visualize the intravascular space and second harmonic generation (SHG) to visualize fibrillar collagen clearly showed a number of pathological changes in AS compared with control healthy mice. These included enlarged aberrant glomerular capillaries and albumin leakage from glomeruli into the tubular fluid already in early-stage AS mice (Figure 1, A and B). In addition, as a clear sign of disease progression, more severe pathologies were present in late-stage AS mice, including enormous and irregular distension of glomerular capillaries, robust albumin leakage through the GFB, the presence of tubular protein casts, focal interstitial fibrosis, and a heterogenous mixture of collapsing, sclerotic and seemingly normal glomeruli (Figure 1C).

A closer look at the glomerular microcirculation in AS revealed that the distension of microvessels (capillary diameter >20 μm vs. ~7 μm in controls) was especially pronounced at the afferent end of glomerular capillaries and included the terminal portion of the afferent arteriole (AA) (Figure 1, D and E). In addition, localized bulging (aneurysm) of glomerular capillaries and microthrombi and albumin-excluding dark (unlabeled) single cells sticking to the capillary wall and blocking capillary blood flow were commonly observed (Figure 1, E and F). Quantitative analysis of glomerular microcirculatory parameters confirmed the significant, progressive increases in glomerular (Figure 1G), AA (Figure 1H), and glomerular capillary diameters (Figure 1J) as well as in glomerular albumin leakage (Figure 1K) in early-and late-stage AS mice compared with controls, while the efferent arteriole diameter was reduced (Figure 1I). The progressive deterioration of these microvascular features correlated with not only GFB functional decline (Figure 1K), but also with reduced podocyte number. This was quantified on the basis of p57^+^ glomerular cell number and glomerular endothelial injury, as indicated by the increased density of plasmalemma vesicle–associated protein (PLVAP), which has been used as a marker of GEC injury and remodeling (11, 12) (Figure 1, L–P). Increased glomerular tuft PLVAP expression (typical of early-stage AS mice at 2 months of age) preceded the reduction in podocyte number in AS mice (typical of late-stage AS mice at 5 months of age) (Figure 1, O and P).

### Increased glomerular endothelial glycocalyx thickness and immune cell homing.

**Figure 2:**
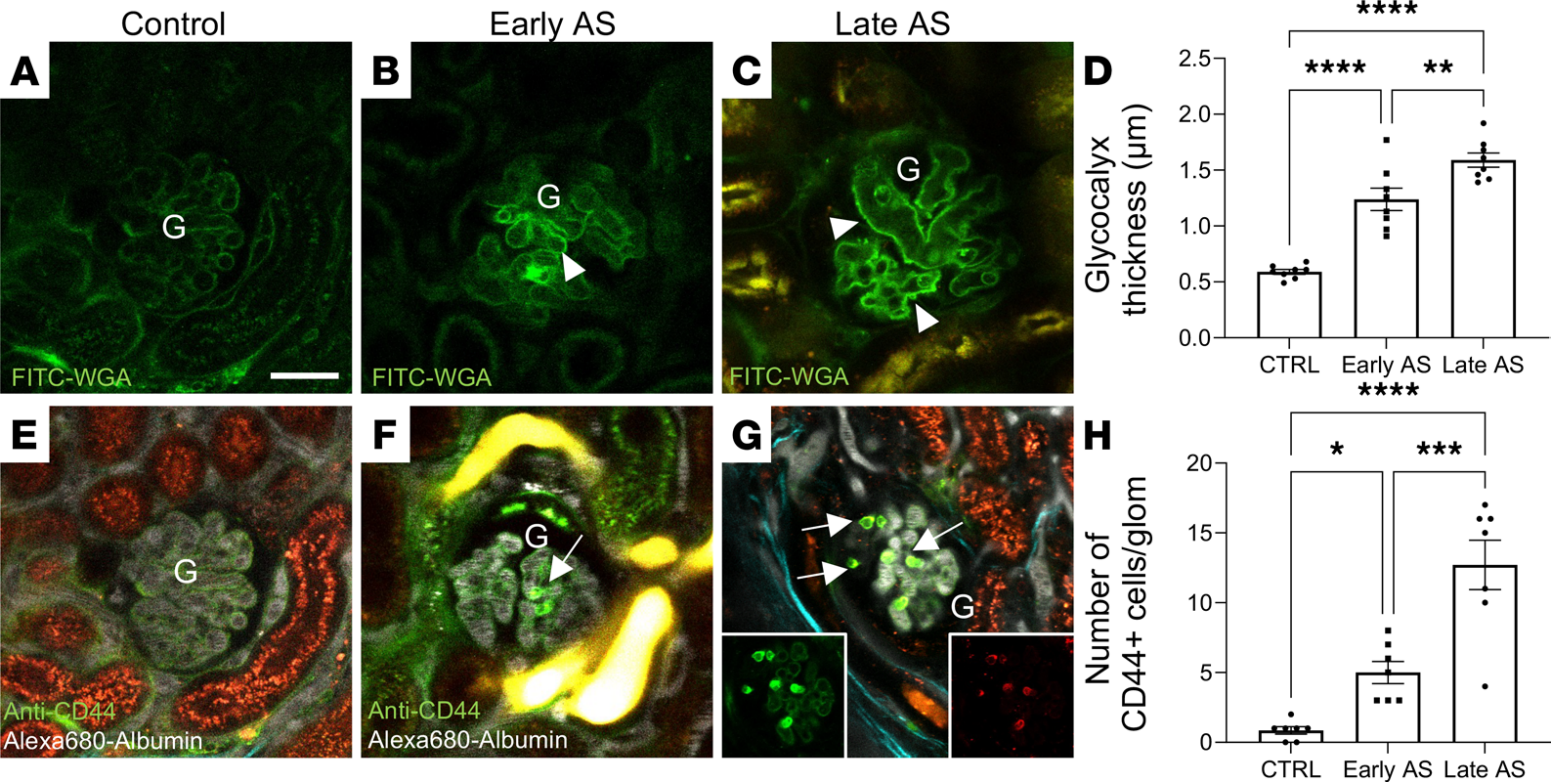
Intravital MPM imaging of glomerular endothelial and immune cell features in control and AS mice. Glomerular endothelial (**A**–**D**) and immune cell features (**E**–**H**). The endothelial surface layer (glycocalyx) was labeled with FITC-WGA lectin (green; linear pattern along capillary lumen), while circulating immune cells were identified with anti-CD44–Alexa Fluor 488 antibodies (green; round single cell pattern), both injected i.v. (**A–D**) Representative images and statistical summary of the significantly increased FITC-WGA intensity and thickness of the GEC glycocalyx (arrowheads) compared with controls (**A**), in a segmental pattern in early-stage AS mice (**B**), and in a global, homogenous pattern in late-stage AS mice (**C**). (**E–H**) Representative images and statistical summary of the increased number of CD44^+^ immune cells (arrows) in control and AS mice. Tissue autofluorescence is shown in orange. Note the lack of CD44^+^ cells in peritubular capillaries. (**G**) An overlay of anti-CD44–Alexa Fluor 488 (green; left inset) and anti-CD–Alexa Fluor 594 antibody labeling (red; right inset). G, glomerulus. Scale bar: 20 μm (throughout, insets represent 3-fold reduction in full frame). Data are shown as the mean ± SEM. *P < 0.05, **P < 0.01, ***P < 0.001, ****P < 0.0001, using 1-way ANOVA with Tukey’s multiple-comparison test. Data points represent the average of multiple measurements/mouse for *n* = 8 mice in each group.

To identify and analyze GECs and the albumin-excluding cells observed in the glomerular capillary plasma in more detail, the endothelial glycocalyx and endogenous immune cells were identified using intravenous injection of FITC-labeled wheat germ agglutinin (FITC-WGA) and Alexa Fluor 488–conjugated anti-CD44 antibodies, respectively. Intravital MPM imaging revealed significantly more intense FITC-WGA labeling and thickening of the GEC luminal glycocalyx in AS mice compared with controls (Figure 2, A–D). This alteration of GECs was observed first in a heterogenous, segmental-like pattern in early-stage AS mice (Figure 2B), but it was seen in a more substantial and homogenous fashion throughout the entire glomerulus in late-stage AS mice (Figure 2C).

In addition, fluorescent labeling of endogenous, circulating immune cells found no or only a few CD44^+^ cells in control glomeruli (Figure 2E) but a significantly increased number in AS glomeruli (Figure 2, F–H). The homing of CD44^+^ cells in the AS kidneys was specific to the glomerular microcirculation, while only a few of these cells were found in peritubular capillaries (Figure 2G). Colabeling with Alexa Fluor 594–conjugated anti-CD3 antibodies showed an almost complete overlap of the CD44^+^ and CD3^+^ cell populations, suggesting that the majority of these immune cells were activated T cells. As with the above microcirculatory parameters, there was a progressive increase in both GEC glycocalyx thickness and CD44^+^ immune cell glomerular homing in early- and late-stage AS mice (Figure 2H).

### Effects of hyaluronidase and ACEi treatment.

**Figure 3:**
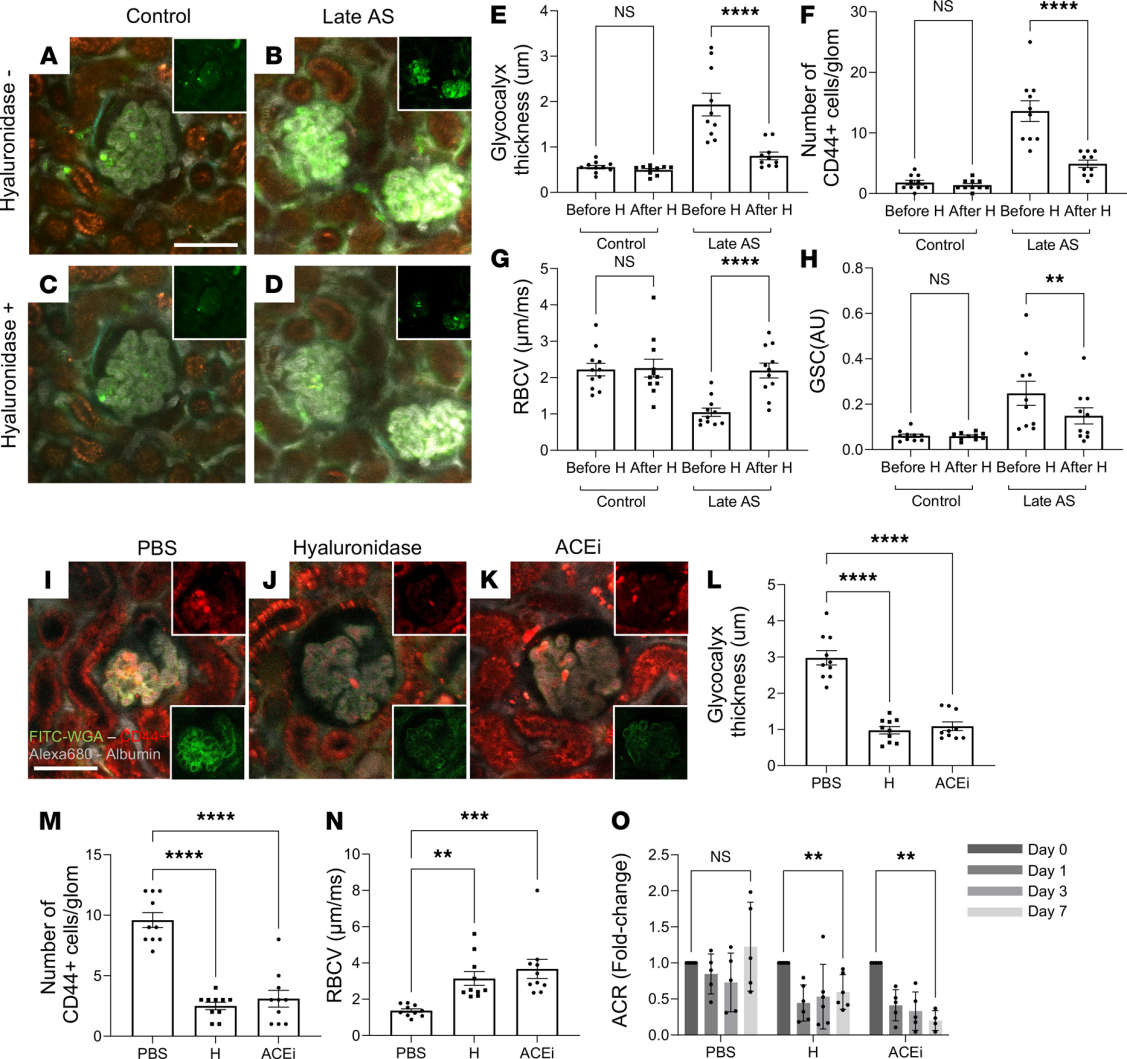
Time-lapse in vivo MPM imaging of the effects of hyaluronidase and ACEi treatment in late-stage AS mice. (**A–H**) Acute hyaluronidase (H) treatment in control (**A** and **C**) and late-stage AS mice (**B** and **D**). Plasma was labeled with albumin–Alexa Fluor 680 (gray), glycocalyx with FITC-WGA lectin (green; linear pattern), and immune cells with anti-CD44–Alexa Fluor 488 antibodies (green; round cell pattern). (**A–D**) Projection 5-minute time-lapse images (overlay of plasma albumin, gray; glycocalyx and immune cell labeling, green; tissue autofluorescence, orange; insets show glycocalyx and CD44^+^ cells separately) of the glomerular microenvironment before (**A** and **B**) and within 1 hour of i.v. H (50 U) injection (**C** and **D**) in control (**A** and **C**) and late-stage AS (**B** and **D**). Note the high glomerular capillary albumin intensity in late-stage AS mice and its reductions after H treatment (**B** and **D**), indicating improved blood flow (flow of nonfluorescent red blood cells [RBC] rather than only the highly fluorescent plasma; Supplemental Video 1). (**E–H**) Summary of the effects of H treatment in control and late-stage AS mice on GEC glycocalyx thickness (**E**), CD44^+^ cell number per glomerulus (**F**), glomerular capillary blood flow (RBC velocity; **G**), and glomerular albumin leakage (albumin GSC; **H**). (**I–O**) Chronic treatment with H, ACEi, or control PBS for 1 week in late-stage AS mice. Projection 5-minute time-lapse images, as in **A–D** (insets show glycocalyx (green) and CD44^+^ cells (red) separately), in control vehicle (PBS) (**I**), H (**J**), and ACEi treatment groups (**K**). (**L–O**) Summary of the effects of control PBS, H, or ACEi treatment on GEC glycocalyx thickness (**L**), CD44^+^ cell number per glomerulus (**M**), glomerular capillary blood flow (**N**), and albuminuria (albumin/creatinine ratio [ACR]; **O**). Data are shown as the mean ± SEM. **P < 0.01, ***P < 0.001, ****P < 0.0001, using 1-way ANOVA followed by Tukey’s or Šidák’s multiple-comparison test. Scale bar: 50 μm. Data points represent the average of multiple measurements/mouse (**O**) or from 2 glomeruli/mouse (**E–N**); *n* = 5–6 mice in each group.

To functionally test the pathogenic role of the excess GEC glycocalyx in AS and the effects of its therapeutic targeting, we first performed acute intravenous injections of the glycocalyx-degrading enzyme, hyaluronidase (H), during continuous time-lapse MPM imaging of glomeruli in control and late-stage AS mice. Within 1 hour of injection, acute H treatment (50 U) in AS mice significantly reduced the excess GEC glycocalyx thickness to approximately control levels (Figure 3, B, D, and E). Simultaneously, H treatment substantially decreased the glomerular homing of CD44^+^ cells, and the immune cells returned to the circulation (Figure 3, A–F, and Supplemental Video 1; supplemental material available online with this article; https://doi.org/10.1172/jci.insight.152676DS1). H treatment in control mice had no significant effects on these parameters (Figure 3, A and C). Importantly, within this short time window (within 1 hour), the removal of excess GEC glycocalyx and CD44^+^ cell homing by H injection normalized glomerular capillary blood flow (Figure 3G) and substantially improved GFB barrier function, as measured by the reduced glomerular albumin leakage (Figure 3H).

We next performed chronic administration of H, enalapril, or control vehicle for 1 week in late-stage AS mice. H and ACEi treatment equally reduced GEC glycocalyx thickness (Figure 3, I–L) and the number of immune cells per glomerulus (Figure 3, I–K and M), improved glomerular blood flow (Figure 3N), and reduced albuminuria (Figure 3O) compared with control treatment with vehicle (PBS).

### Renal histology features of mouse and human AS.

**Figure 4:**
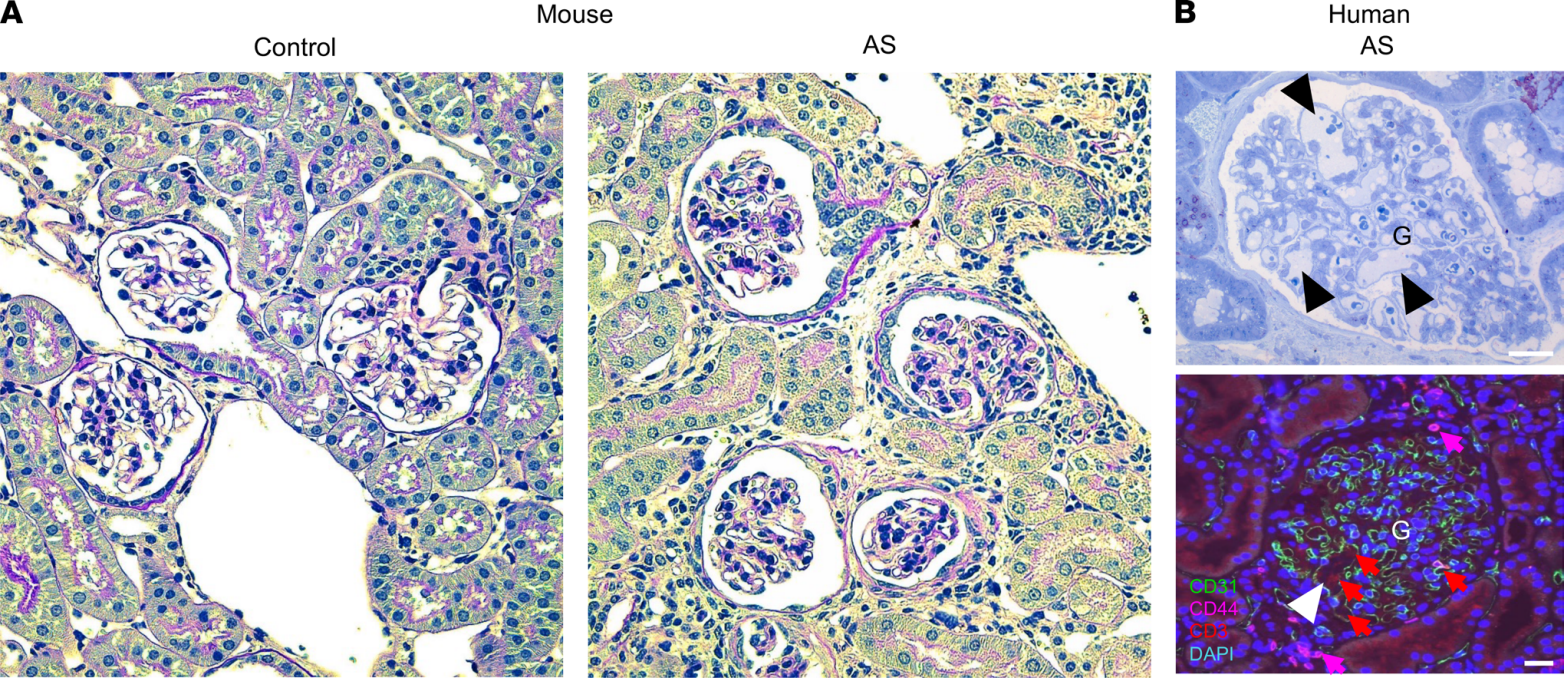
Histological features of mouse and human kidney tissue AS sections. (**A**) Periodic acid–Schiff–stained (PAS-stained) thin sections of the kidney tissue from same mice that were used for intravital imaging. Note the lack of severely distended glomerular capillaries in either control or AS tissues. (**B**) Representative semithin section with toluidine blue staining (top) of 5 of 34 samples from patients with AS and a paraffin section with immunofluorescence multilabeling (bottom) for CD31 (green), CD44 (purple) and CD3 (red). Note the presence of distended glomerular capillaries (arrowheads) and CD44^+^ and CD3^+^ immune cells in the glomerular capillary lumen (red and purple arrows, respectively) in these specific human AS sections. Nuclei are stained blue with DAPI. Scale bars: 20 μm (all images).

Finally, to confirm whether the observed glomerular structural alterations can also be found in mouse or human renal histological sections, we performed histological staining of mouse kidney tissue and renal biopsy specimens from patients with AS (Figure 4, A and B). Glomerular capillary distension was not observed in histological paraffin sections of the same mice that showed the phenotype of capillary aberrations in vivo with MPM (Figure 4A). A preliminary qualitative analysis of kidney sections from humans with AS (i.e., from routine diagnostic biopsies) detected the presence of CD3^+^ and CD44^+^ immune cells in glomeruli; however, distended capillaries were rarely seen (Figure 4B). Only 5 patient samples showed some minor capillary irregularities of 34 patients with AS examined.

## Discussion

This study applied an unbiased intravital imaging approach using MPM of the local kidney tissue microenvironment to search for direct visual clues concerning the major drivers of AS pathology. The key findings were the clear signs of progressive glomerular endothelial and immune cell activation already in an early phase of disease pathogenesis, and included severely distended glomerular capillaries, aneurysms and microthrombi, increased glomerular endothelial glycocalyx thickness and immune cell homing, and albumin leakage through the damaged GFB. Complementary histological analysis identified and confirmed the increased expression of the GEC injury marker PLVAP (11) in early- and late-stage AS mice and reduced podocyte number in late-stage AS mice, consistent with endothelial injury preceding podocyte damage. Interestingly, glomerular capillary distension was not observed in histological paraffin sections of the same mice that showed the phenotype of capillary aberrations in vivo with MPM. While the presence of immune cells in glomeruli was detected in fixed human AS kidney sections, suggesting the translatability of at least some of our findings to human disease, distended capillaries were rarely seen in classic histological analysis. Because the newly acquired visual clues pointed to endothelial activation and excess glycocalyx as the main early culprits, a potentially new mechanism-based therapeutic approach was devised and used both as acute and chronic treatment, with the glycocalyx-degrading enzyme, H, in our animal model for initial proof of concept. This treatment had no obvious adverse effects but led to improvements in the structure and function of the GFB and glomerular capillary blood flow and reduced albuminuria and tissue inflammation. Treatment for 1 week with H or the gold-standard ACEi therapy reduced to a similar extent GEC glycocalyx thickness and immune cell homing and improved glomerular blood flow and proteinuria, further suggesting the importance of these endothelial alterations in the pathogenesis of CKD in AS. Altogether, this study helps to improve our mechanistic understanding of AS pathogenesis and opens the way for the development of new, mechanism-based therapeutic approaches for AS.

The deposition of aberrant collagen by podocytes that leads to defective GBM, podocyte loss, and GFB dysfunction is the currently accepted mechanistic paradigm of AS pathogenesis and disease progression (2). As reported recently, podocyte damage and loss during disease progression is evident and is accompanied by increased proteinuria in both humans and the same AS mouse model that was used in this study. These mice succumb to kidney failure and die at around 6 months of age (3, 4, 12). However, our findings suggest that AS may not be simply a podocyte-driven disease. Our recent work established the role of GECs in early-stage AS (3), and GEC damage is known to precede podocyte loss and proteinuria development in multiple renal pathologies including AS (4, 5). Located on the other side of the defective GBM, GEC injury could be secondary to altered podocyte crosstalk, e.g., via altered VEGF signaling (4). However, GECs may well also be affected by the AS microenvironment independently of podocyte effects.

Conventional histology using paraffin sections has not been able to show distended glomerular capillaries in AS mice or patients with AS, likely due to technical constraints (e.g., normal or elevated capillary pressure was lost during fixation). In contrast, intravital MPM imaging in this work found robust evidence of distension and aneurysms of glomerular microvessels, primarily the AA and the afferent end of capillaries, where the intravascular pressure is known to be the highest (~60 mmHg) in the glomerular microcirculation (Figure 1, E and F). This finding emphasizes the great advantage and need of using intravital MPM for studying AS in the intact living kidney. The observed capillary aberrations are consistent with a weakened GBM contributing to podocytes’ and mesangial cells’ lost capability to counter the expansile forces of high capillary pressures. This will only worsen as glomerular sclerosis and loss raise the target single-nephron glomerular filtration rate and hence transcapillary hydrostatic pressures for the remnant glomeruli, which may be why in vivo AA and capillary widening worsens with disease progression. The presence of elevated biomechanical strain of the 3 glomerular cell types, podocytes and endothelial and mesangial cells, in AS has been predicted and deduced before (13, 14). Based on the visualization of distended glomerular capillaries and AA in vivo in this study, we speculate that the findings were driven by increased mechanical load.

The second key finding of this study was the substantially (~3-fold) increased glomerular endothelial glycocalyx thickness (Figure 2, A–D) that was likely due to the activation of GECs in AS. The heterogeneity of FITC-WGA labeling, i.e., intense labeling of significantly thickened glycocalyx in some but not all glomerular capillary segments in early-stage AS (Figure 2B), is consistent with our recent finding on the identification of distinct GEC subpopulations and altered expression of GEC glycocalyx components in this condition (3). However, the increased rather than decreased glycocalyx thickness that accompanied glomerular albumin leakage (Figure 1, B–F and K) was an unexpected finding, because according to the current paradigm, the glomerular endothelial glycocalyx is an important permselectivity factor for the GFB. Accordingly, kidney injury models are usually characterized by glomerular endothelial glycocalyx shedding causing albumin leakage, as was also demonstrated in our previous MPM imaging work (15, 16). One exception was lupus nephritis, which was similarly associated with increased GEC glycocalyx as reported recently (10). These findings suggest that the alterations in GEC glycocalyx per se are not always directly related to proteinuria development (either increased or decreased glycocalyx can be pathogenic) and underscore the importance of additional pathogenic factors and the need for comprehensive functional analysis (such as analysis of immune cell–mediated local inflammation).

The third phenotypic feature of AS found in this imaging study was the preferential glomerular homing of immune cells (Figure 2, E–H) that were identified as activated T cells (Figures 2 and 3). This finding is consistent with the well-established role of tissue inflammation in AS pathogenesis, although cytokines released by resident kidney cell types (17), and monocytes and lymphocytes resident in the tubulointerstitium (18, 19) rather than in glomeruli, have been thought to be the main mechanisms. The preferential glomerular homing of T cells fits well with the excessive GEC glycocalyx, which was our other main finding discussed above. Among the many molecular mechanisms of immune cell homing, the binding of the CD44 receptor (highly expressed in T cells) to its main ligand hyaluronic acid (a major component of GEC glycocalyx) is one such mechanism (20, 21) that is likely at play in the glomerular immune cell homing observed in our AS mouse model. The effect of H treatment removing the excess GEC glycocalyx as well as depleting glomerular immune cells (Figure 3 and further discussed below) is supportive of this notion. The key role of this same CD44–hyaluronic acid interaction in T cell homing was demonstrated recently in lupus nephritis (10).

Enzymatic removal of the excess GEC glycocalyx by H treatment and its beneficial effect on blocking glomerular immune cell homing was directly confirmed by our MPM imaging approach using either acute or chronic treatment (Figure 3). In acute H treatment, this functional test provided unequivocal evidence supporting the primary role of GECs in AS pathogenesis and also a proof of concept for the efficacy of therapeutically targeting the excess GEC glycocalyx in AS (Figure 3, A–H). Glomeruli were evaluated within 1 hour after H administration based on previous reports that H’s effect on capillary glycocalyx was maximal at this time point (22, 23). Importantly, chronic H treatment for 1 week had similar effects: it reduced the excess glomerular endothelial glycocalyx, almost completely blocked immune cell homing, improved glomerular capillary blood flow, and significantly reduced but did not completely eliminate albumin leakage through the GFB (Figure 3, I–O). These findings suggest that the protective effects of H are sustained over longer time periods, further confirming our recent findings in lupus nephritis (10). In addition, these results strongly suggest the key and primary importance of GECs rather than podocytes or mesangial cells in early-stage AS pathogenesis, although contributions of these other glomerular cell types and the defective GBM to AS are still acknowledged (2, 6, 13, 17). The residual GFB albumin leakage or albuminuria after H treatment (Figure 3, H and O) is also consistent with the classic role of GBM and podocyte mechanisms in AS. Although it has been established that H injection does not affect glomerular charge selectivity or permeability of macromolecules (24), the improvement in glomerular albumin leakage in response to glycocalyx removal in this study seems paradoxical, considering that GEC glycocalyx degradation has previously been linked to pathology development (15, 16). However, the removal of excess glycocalyx and consequent blockade of CD44–hyaluronic acid interaction, led to the depletion of glomerular immune cells, likely decreasing local tissue inflammation, the presumed reason for the protective effects of H treatment. Interestingly, H treatment produced similar effects via these same mechanisms in a model of lupus nephritis, raising the possibility that GEC glycocalyx accumulation and its pathogenic effects are more widespread than previously thought (10). Importantly, these results also provided proof of concept for the relevance of a glycocalyx-targeting therapy for AS. This approach may open the way for the future development of more specific and upstream mechanism-based treatments in contrast to the currently used standard-of-care nonspecific therapy with renin-angiotensin system inhibitors or even some newer therapeutic options (6, 25). The antiinflammatory effect of blocking immune cell homing with H treatment is consistent with the well-known major role of tissue inflammation in AS (2, 6). Interestingly, chronic treatment with gold-standard ACEi therapy for 1 week similarly reduced GEC glycocalyx and immune cell homing and improved glomerular blood flow and proteinuria (Figure 3, I–O). These results shed light on possible novel modes of action for the protective effects of ACEi therapy (reduced endothelial stress and glycocalyx output) and further suggest the relevance of glycocalyx-targeting therapies and the importance of the observed endothelial alterations in AS.

In summary, this study identified possibly new structural and functional disease phenotypes of AS based on an intravital MPM imaging approach. Our findings strongly suggest that, while all 3 glomerular cell types are involved, GECs may play the primary role in AS disease initiation and early progression. GEC glycocalyx targeting may be a new mechanism-based therapeutic approach identified in this study for early-stage AS.

## Methods

### Mice.

Transgenic Alport mice (B6.Cg-Col4a5tm1Yseg/J, stock 006283) on a C57BL/6 background, as developed previously (26), were purchased from The Jackson Laboratory. AS mouse colonies were bred and maintained at the Children’s Hospital Los Angeles in specific pathogen–free quarters according to a homozygous/hemizygous breeding scheme. Early-stage (2 months old) and late-stage (5 months old) male AS and healthy age-matched mice were used in this study.

### Intravital MPM.

Under continuous anesthesia (isoflurane 1%–4% inhalant via nose cone), the left kidney was exteriorized through a flank incision, and the animals were placed on the stage of an inverted microscope with the exposed kidney placed in a coverslip-bottomed chamber bathed in normal saline, as described previously (7, 8). Body temperature was maintained with a homeothermic blanket system (Harvard Apparatus). Alexa Fluor 680–conjugated bovine serum albumin (Thermo Fisher Scientific) was administered i.v. by retro-orbital injection to label the circulating plasma (30 μL i.v. bolus from 10 μg/ml stock solution). The images were acquired using a Leica SP8 DIVE multiphoton confocal fluorescence imaging system with a ×40 Leica water-immersion objective (NA 1.1) powered by a Chameleon Discovery laser at 960 nm (Coherent) and DMI8 inverted microscope’s external Leica 4Tune spectral hybrid detectors (emission at 500–530 nm for FITC and Alexa Fluor 488, at 675–750 nm for Alexa Fluor 680, and at 475–485 nm for detecting second harmonic generation) (Leica Microsystems). The potential toxicity of laser excitation and fluorescence to the cells was minimized by using a low-laser power and high scan speeds to keep total laser exposure as minimal as possible. The usual image acquisition (12 bit, 512 × 512 pixel) consisted of only 1 *Z*-stack tissue volume (*xyz* with 1 μm steps within <2 min) or time-lapse (*xyt* every 5 s for up to 15 min) collection per glomerulus, which resulted in no apparent cell injury. Image analysis and fluorescence intensity measurements were assessed by LAS X software (3.6.0.20104, Leica Microsystems).

### Glomerular hemodynamics.

Alexa Fluor 680–conjugated bovine serum albumin was used to label the circulating plasma and the negative labeled (albumin-excluding) red blood cells. Glomerular capillary blood flow was evaluated based on measurement of red blood cell velocity using line (*xt*) scans of capillary lumen, as described previously (8). GFB function was evaluated based on measurement of albumin leakage into the Bowman’s space (glomerular sieving coefficient, GSC of albumin) (8, 27). Regions of interest were drawn in glomerular capillary plasma and the Bowman’s space, and image analysis was performed as described previously (16, 28).

### Endothelial glycocalyx (GEC glycocalyx) imaging.

FITC-WGA lectin (Triticum vulgaris; L4895, MilliporeSigma), administered via retro-orbital sinus at 2 μg/g body weight was used to visualize the entire glomerular endothelial glycocalyx. The FITC-WGA lectin–positive region of the glomerular endothelial capillary surface was visible immediately after injection. FITC-WGA lectin fluorescence intensity and thickness were evaluated before and 10 minutes after H treatment (50 U). Quantification of glycocalyx thickness was performed on capillary wall line profiles by calculating the width of FITC-WGA signal at half-maximum fluorescence intensity as described previously (15, 16).

### In vivo labeling and analysis of endogenous CD3^+^CD44^+^ T cell homing.

Alexa Fluor 594–labeled anti-CD3 antibody (BioLegend) and Alexa Fluor 488–labeled anti-CD44 antibodies (BioLegend) were used to detect activated T cells and to directly and quantitatively visualize their glomerular intravascular homing in WT and AS mice using time-lapse MPM as described recently (10). These antibodies were administered via retro-orbital sinus each at dose of 30 μl. Positively labeled cells could be seen clearly immediately after antibody injection. The number of CD3 and CD44 double-positive cells in entire glomeruli were counted in 3D volume (*xyz*) images before and after H treatment. Multiple glomeruli per each mouse were analyzed to avoid sampling errors. In some experiments, time-lapse imaging of the same *z* plane was performed for 5–10 minutes, and single projection images of the entire *xyt* sequence were generated to illustrate the dynamics and magnitude (CD3^+^CD44^+^ cell density) of immune cell surveillance.

### Kidney tissue histology.

Immunofluorescence detection of proteins was performed as described previously (29). Briefly, kidneys were perfused and fixed in 4% PFA for 2 hours at room temperature, embedded in paraffin, and sectioned 8 μm thick. For antibody stains, slides were washed in 1X PBS. For antigen retrieval, heat-induced epitope retrieval with sodium citrate buffer (pH 6.0) or Tris-EDTA (pH 9.0) was applied. To reduce nonspecific binding, sections were blocked with normal serum (1:20). Primary and secondary antibodies were applied sequentially overnight at 4°C and 2 hours at room temperature. Primary antibodies and dilutions were as follows: anti-p57 (1:100, Abcam), PLVAP (1:100, Bio-Rad), and CD31 (1:100, Cell Signaling Technology). Alexa Fluor 488–, 594–, and 647–conjugated secondary antibodies were purchased from Invitrogen. Slides were mounted by using DAPI-containing mounting media (VectaShield, Vector Laboratories). Sections were examined with Leica TCS SP8 (Leica Microsystems) confocal/multiphoton laser scanning microscope systems as described previously (29–31). In addition, Periodic acid–Schiff–stained mouse kidney sections (2 μm thick) were imaged and analyzed using Leica LAS EZ 3.4 software.

### H and ACEi treatment.

In acute studies, control and AS mice were given i.v. via retro-orbital sinus a 0.02 ml bolus of either vehicle (PBS) or H (50 U, MilliporeSigma, H3506). The thickness of GEC glycocalyx (width of FITC-WGA signal), the number of CD3^+^ and CD44^+^ cells, glomerular capillary blood flow, and albumin GSC in the same glomerulus were measured before and within 1 hour of H treatment. In chronic studies, H (200 U, as above) was given i.v. every other day for a total of 3 times, as described recently (10). The ACEi enalapril was administered at a dose of 10 mg/day/mouse via drinking water (150 mg/L), as described previously (32). Animals were treated with H, ACEi, or vehicle (PBS given i.v.) for 1 week followed by intravital MPM imaging and tissue harvest as described above. The albumin/creatinine ratio was measured from spot urine samples collected on days 0, 1, 3, and 7 using ELISA (10).

### Cohort of patients with AS and renal histology.

AS diagnosis was made at the Department of Nephropathology, Friedrich-Alexander University, Erlangen-Nürnberg, based on patient family history, clinical data (hematuria, proteinuria), light microscopy, negative immune histology, and, more specifically, electron microscopy of routine kidney biopsy specimen as described recently (33). For this study, archived toluidine blue–stained semithin sections of routine renal biopsies of 34 patients with AS were examined. Immunofluorescence labeling of CD31^+^, CD3^+^, and CD44^+^ cells was performed on residual PFA-fixed archived biopsies as described above for mouse kidney tissue sections.

### Statistics.

Data are reported as mean ± SEM, with *n* referring to the number of animals studied. One-way ANOVA with Šidák’s (for paired experiments) or Tukey’s multiple-comparison test was performed using GraphPad Prism 8. *P* < 0.05 was considered significant.

### Study approval.

All animal protocols were approved by the Institutional Animal Care and Use Committee at the Children’s Hospital Los Angeles and at the University of Southern California. The studies of residual archived human renal biopsies were conducted according to the principles of the Declaration of Helsinki and were approved by the ethics committee of the Friedrich-Alexander University Erlangen-Nürnberg (approval 251_18 B).

## Author contributions

GG, UNS, AI, XH, SDS, SS, and KA conducted the experiments. GG, LP, KVL, and JPP designed the study and wrote the paper.

## Supplementary Material

Supplemental video 1

## Version 1 11/18/2021

In-Press Preview

## Version 2 01/11/2022

Electronic publication
